# Serological and PCR investigation of *Yersinia pestis* in potential reservoir hosts from a plague outbreak focus in Zambia

**DOI:** 10.1186/s13104-017-2667-9

**Published:** 2017-07-28

**Authors:** S. S. Nyirenda, B. M. Hang’ombe, E. Mulenga, B. S. Kilonzo

**Affiliations:** 1 Central Veterinary Research Institute, Ministry of Fisheries and Livestock, Lusaka, P.O. Box 33980, Zambia; 2 School of Veterinary Medicine, University of Zambia, Lusaka, Zambia; 3 Department of Veterinary Microbiology and Parasitology, Sokoine University of Agriculture, Morogoro, Tanzania

**Keywords:** *Yersinia pestis*, Rodents, Shrews, Dogs, Cats, Fleas, ELISA, PCR

## Abstract

**Background:**

Plague is a bacterial zoonotic disease, caused by *Yersinia pestis*. Rodents are the natural hosts with fleas as the vehicle of disease transmission. Domestic and wild dogs and cats have also been identified as possible disease hosts. In Zambia, plague outbreaks have been reported in the Southern and Eastern regions in the last 20 years. Based on these observations, *Y. pestis* could possibly be endemically present in the area.

**Methods:**

To substantiate such possibility, sera samples were collected from rodents, shrews, dogs and cats for detection of antibodies against Fraction 1 gene (Fra1) of *Y. pestis* while organs from rodents and shrews, and fleas from both dogs and rodents were collected to investigate plasminogen activator gene (*pla gene*) of *Y. pestis* using ELISA and PCR respectively.

**Results:**

A total of 369 blood samples were collected from domestic carnivores, shrews and domestic and peri-domestic rodents while 199 organs were collected from the rodents and shrews. Blood samples were tested for antibodies against Fra1 antigen using ELISA and 3% (5/165) (95% CI 0.99–6.93%) dogs were positive while all cats were negative. Of 199 sera from rodents and shrews, 12.6% (95% CI 8.30–17.98%) were positive for antibodies against Fra1 using anti-rat IgG secondary antibody while using anti-mouse IgG secondary antibody, 17.6% (95% CI 12.57–23.60%) were positive. PCR was run on the organs and 2.5% (95% CI 0.82–5.77%) were positive for plasminogen activator gene of *Y. pestis* and the amplicons were sequenced and showed 99% identity with *Y. pestis* reference sequences. All 82 fleas collected from animals subjected to PCR, were negative for *pla* gene. The specific rat-flea and dog-flea indices were 0.19 and 0.27 respectively, which were lower than the level required to enhance chances of the disease outbreak.

**Conclusions:**

We concluded that plague was still endemic in the area and the disease may infect human beings if contact is enhanced between reservoir hosts and flea vectors. The lower specific rodent-flea Indices and absence of *Y. pestis* in the potential vectors were considered to be partly responsible for the current absence of plague outbreaks despite its presence in the sylvatic cycle.

## Background

Plague is a bacterial zoonotic disease, which infects both human beings and animals especially rodents, caused by a gram-negative bacterium known as *Yersinia pestis*. Other animals which are occasionally infected and can serve as carriers and consequently act as a source of infection to humans include domestic and wild dogs, cats, other small carnivores and camels [1]. The disease is mainly transmitted from rodents to other mammals by the bite of an infectious flea. The bacterium infects human beings through the bite of an infected flea or by direct contact with contaminated materials from an infected animal [2]. Transmission to animals such as cats and other small carnivores can also occur through bites by fleas escaping from infected rodents caught by such predators or through consumption of such rodent carcases [3].

In Zambia, there have been three outbreaks of human plague in three different zones that are far apart and a number of cases were recorded. The outbreak in North-Western region occurred in December 1993 [4], while in Southern zone, the outbreak occurred in December 1996 [5]. The recorded outbreak in Eastern zone occurred in January 2001, where 436 human cases of bubonic plague with 11 deaths were reported and in 2007, 32 cases were involved [6–8]. All these outbreaks were attributed to the possible movement of rodents from burrows to the nearby villages as a result of heavy rains which flooded these burrows. Plague has been known to occur following a period of total silence as has been observed in India, which experienced a large outbreak in 1994 after 30 years [9], in Algeria, cases were reported in 1940 after a 50 year period of quiescence [10–12] and in Libya, after 25 years, only to have cases in 2009 [13, 14]. The purpose of this study was to assess the role of these hosts, found or living close to humans, as indicators of plague occurrence in the area using molecular and serological tools in a previously plague outbreak area.

## Methods

### Animal sampling

Blood sampling from domestic dogs and cats was done following both written and verbal consent from the owners through their village headmen and the Veterinary camp officer. Animals were sampled from villages in the Eastern region of Zambia (14°12′43.25″S and 31°45′35.95″E), where there was an outbreak of plague in 2001 and 2007 [6, 8]. This region is situated on a plateau area whose altitude is about 1087 metres above sea level. The study was conducted between September 2012 and August 2013.

Trapping of domestic and peri-domestic rodents was done using Shermans live traps (50 × 65 × 157 mm) and wire meshed cages (145 × 100 × 230 mm) (Hoga-lab, Kyoto, Japan) in the nearby bushes and in houses respectively [15]. Each captured rodent or shrew was put in a bag containing cotton wool soaked with 90% diethyl ether to anaesthetise both the host and the ectoparasites. It was then transferred to a silver basin and brushed with a tooth brush to remove fleas and other ectoparasites. Fleas which dropped into the basin and those that remained attached to the animal skin and fur were gently removed with a pair of fine forceps into small vials containing 70% ethanol.

Cardiac blood was collected from each rodent or shrew using a 2 ml syringe and a 21G needle and left at room temperature for serum separation. The separated serum was collected into sterile vials and preserved at −20 °C until required for use.

Each rodent or shrew was aseptically dissected and organs (spleen, liver, lung, kidney and heart) were collected into sterile vials and stored at −20 °C until required for use.

Blood sampling from domestic dogs and cats was done following consent from the owners. The selected animals were restrained and about 1- to 2-ml blood was collected from the femoral vein into the sterile plain tube and left overnight for serum separation. The serum was transferred into sterile serum vials and stored at −20 °C until required for use. Fleas were also collected from the restrained animal by lying the latter on the white sheet and brushing the host with the cotton wool soaked with 90% diethyl ether to anaesthetise the fleas, and brushed using the appropriate animal brush from head to tail. Fleas on the white sheet were collected into the small vial containing 70% ethanol. Fleas that remained attached to the animal skin and fur were gently removed with a pair of fine forceps into clean vials.

All experimental protocols were reviewed and validated by our Institutional Ad hoc committee for the care and use of animals.

### DNA extraction and PCR detection of *Y. pestis*

The DNA from tissues of rodents was extracted using the DNA extraction kit (ZR genomic DNATM-Tissue MiniPrep Catalog No. D3051) following the manufacturer’s instructions (Zymo Research Irvine, CA, USA). In the case of fleas, these were identified before preparing them for DNA extraction using heat treatment method. The fleas were sorted out according to species collected from the animal and pooled. Each pool containing up to three fleas, was put in Eppendorf tube and 50 µl of Brain–Heart infusion broth (Oxoid, Hampshire, England) was added and triturated with sterile pipettes. The triturated samples were then boiled at 95 °C in Driblock bath (Scinics, Japan) for 10 min and centrifuged at 10,000×*g* for 10 s, after which 10 µl of each sample was collected in a clean eppendorf tube and subjected to PCR testing [5, 16].

Detection of *Y. pestis* was accomplished using the conventional PCR technique. The *Y. pestis* specific primers were used to target the plasminogen activator (*pla*) gene, which is encoded in plasmid pPCP1 of *Y. pestis*. *Yp pla1*: (5′-ATC TTACTT TCC GTG AGA AG-3′) and *Yp pla2*: (5′-CTT GGA TGT TGA GCT TCC TA-3′) corresponding to nucleotides 971–990 and 1431–1450 of the *pla* locus sequence, respectively and detected at 479 bp [17]. PCR was done by Phusion™ flash high fidelity PCR master mix (Finnzymes Oy, Finland). The reactions were performed in a final volume of 10 µl containing 5 µl phusion flash PCR master mix, 0.5 µM of primer sets in a 1 µl volume of each, 1 µl of the template and 2 µl of PCR water. The Piko™ thermal cycler (Finnzymes instruments Oy, Finland) was programmed at 95 °C for 10 s for initial denaturation, followed by 35 cycles consisting of 95 °C for 1 s, 58 °C for 5 s and 72 °C for 15 s. Final extension was given 72 °C for 1 min. The correct sizes of the PCR products/bands were then confirmed by agarose gel electrophoresis and the UV illuminator.

### ELISA testing

The stored sera were removed from the freezer (−20 °C) and left at room temperature to thaw and processed using the protocol as described by Chu [18].

### Data analysis

Data was entered in Excel Microsoft software and analysed by using Epi info™ 7.0.8.0, a computer statistical package from the Centre for Disease Control and Prevention (CDC, GA, USA). The flea population density of *Xenopsylla* spp. on rodents and shrews was measured by using flea percentage incidence index (PII) and specific rat-flea index (SFI) as described by Bahmanyar and Cavanaugh [19]. The PII of each rodent or dog species was calculated by dividing the number found infested with fleas by the total number trapped and the result was expressed as a percentage, whereas the SFI was calculated as the number of fleas per rodent or dog species.

## Results

A total of 199 domestic and peri-domestic rodents and shrews were captured and sampled from 25 villages. The captured species from the study area were *Mastomys natalensis* (36.2%), *Crocidura* spp. (43.2%), *Rattus rattus* (8%), *Saccostomus* spp. (7.5%), *Graphiurus* spp. (2.5%), *Mus* spp. (1%), *Tatera* spp. (1%) and *Acomys* spp. (0.5%). Thirty-seven fleas (*Xenopsylla* spp.*)* were the only fleas collected from the rodents giving a rodent-flea index of 0.19 per animal. The SFI for individual rodents were 0.40, 0.31 and 0.20 for *Mastomys natalensis*, *Rattus* spp. and *Saccostomus* spp. respectively. Likewise, the PII were respectively 22.2, 12.5 and 13.3% for the three rodent species (Table 1).

**Table 1:** Specific flea indices and percentage incidence indices of fleas on rodent species

Animal species	Total number caught	Total number infested	Total number of fleas	PII	SFI
*Mastomys natalensis*	72	16	29	22.2	0.40
*Rattus rattus*	16	2	5	12.5	0.31
*Saccostomus* spp.	15	2	3	13.3	0.20
*Tatera* spp.	2	0	0	0	0
*Graphiurus* spp.	5	0	0	0	0
*Crocidura* spp.	86	0	0	0	0
*Mus* spp.	2	0	0	0	0
*Acomys* spp.	1	0	0	0	0
Total	199	20	37	10	0.19

*PII* percentage incidence index; *SFI* specific flea index

As for the domestic dogs, a total of 165 animals were sampled for blood and fleas collection. The dogs comprised 81 females and 84 males and came from 20 different villages. A total of 45 fleas were collected from the sampled dogs and the flea index on these animals was 0.27. All fleas collected from dogs were *Ctenocephalides canis.* PCR results revealed that all the fleas collected from dogs and rodents were negative for *Y. pestis* DNA *pla* gene and 5 (2.5%) (95% CI 0.82–5.77%) of rodents and shrews had *Y. pestis* DNA detected following amplification of a 479 bp product. These amplicons were sequenced using a 3130 Genetic Analyzer and compared with those available in the GenBank by basic local alignment search tool (BLAST) and were 99% identical with *Y. pestis* reference sequences (Accession: CP009936.1; CP010294.1). The positive animals were from four villages and three (1.5%) were *Mastomys natalensis*, one (0.5%) was *Rattus rattus* and one (0.5%) was *Crocidura* spp. (Table 2).

**Table 2:** Numbers of rodents and shrews positive for *Yersinia pestis* DNA gene

Animal species	Number tested	No. of PCR positive	95% confidence interval (CI)^a^
*Mastomys natalensis*	72	3 (4.2%)	0.87–11.70
*Rattus rattus*	16	1 (6.25%)	0.16–30.23
*Saccostomus* spp.	15	0	
*Tatera* spp.	2	0	
*Graphiurus* spp.	2	0	
*Crocidura* spp.	86	1 (1.2%)	0.03–6.31
*Mus pp*	2	0	
*Acomys* spp.	1	0	
Total	199	5 (2.5%)	0.82–5.77

^a^Clopper–Pearson exact CI method was used

ELISA results indicated that animals from 17 villages in the study area were sero-positive for antibodies against Fraction 1 antigen of *Y. pestis.* Using anti-rat as a secondary antibody, 12.6% (95% CI 8.3–17.98) were positive while using the anti-mouse antibody as a secondary antibody, 17.6% (95% CI 12.57–23.6) were positive for *Y. pestis* antibodies respectively (Table 3). Of the 165 domestic dogs tested 5 (3%) (95% CI 0.99–6.93) were positive for specific antibodies against Fra1 antigen of *Y. pestis* while all the cats were negative.

**Table 3:** ELISA results from rodents and shrews using anti-rat and anti-mouse as secondary antibodies respectively

Animal species	No. of sera tested for antibodies	No. of positives when using anti-rat antibody	95% confidence interval (CI)^a^	No. of positives when using anti-mouse antibody	95% confidence interval (CI)^a^
*Mastomys natalensis*	72	12 (6%)	3.15–10.3	17 (8.5%)	5.06–13.33
*Rattus rattus*	16	2 (1.0%)	0.12–3.58	2 (1.0%)	0.12–3.58
*Saccostomus* spp.	15	2 (1.0%)	0.12–3.58	3 (1.5%)	0.31–4.34
*Tatera* spp.	2	1 (0.5%)	0.01–2.77	0 (0.0%)	
*Graphiurus* spp.	5	1 (0.5%)	0.01–2.77	3 (1.5%)	0.31–4.34
*Crocidura* spp.	86	7 (4.0%)	1.43–7.11	9 (4.5%)	2.09–8.41
*Mus* spp.	2	0 (0.0%)		1 (0.5%)	0.01–2.77
*Acomys* spp.	1	0 (0.0%)		0 (0.0%)	
Total	199	25 (12.6%)	8.3–17.98	35 (17.6%)	12.57–23.6

^a^Clopper–Pearson exact CI method was used

## Discussion

The detection of *Y. pestis* DNA plasminogen activator gene in 5 (2.5%) rodents and shrews from the villages which were involved in the 2007 outbreak of plague can be interpreted to indicate that *Y. pestis* is still present in the area. These animals may have been infected with the bacterium either through flea bites or from soil contamination as the bacterium can persist in the soil for substantial periods of time [20, 21]. The fact that *Mastomys natalensis* had the highest infection rate as compared to the house rats and *Crocidura* spp. is consistent with observations by Kilonzo et al. [22]. All the four villages, where the pla gene of *Y. pestis* was detected, were involved in the 2007 outbreaks of plague [7]. However, neither of the villages was affected by the 2001 outbreak of the disease. The sequencing results also indicated that *Y. pestis* circulating in the area was similar to the bacterium which has caused the disease in other parts of the world (Reference sequence accession: CP009936.1; CP010294.1).

The negative PCR results on *Xenopsylla* spp. fleas collected from rodents and shrews suggests that chances of transmitting the bacterium from rodents and shrews to other mammals including humans through flea bite were minimal or non-existent during the time of this study. This observation is probably attributable to the fact that specimen collections were done at a time of disease quiescence, when the bacterium may be lower in the reservoirs. Clover et al. [23] similarly reported that when there is no plague disease outbreak, most fleas become negative for *Y. pestis* DNA.

Despite the presence of *Y. pestis* DNA in rodents and shrews, there was no indication of the outbreak of disease probably due to the partly very low population of vector fleas (*Xenopsylla* spp.) whose individual rat-flea index ranged between 0.0 and 0.4 among the rodents. Such indices are too low to institute a risk of transmitting the disease outbreak. However, the study indicated that rodents and shrews are suitable hosts/carriers of *Y. pestis* in the area and may serve as sources of plague outbreaks if and when climatic conditions become favourable to the main hosts and vectors [24].

The ELISA results further revealed that *Y. pestis* antibodies were present in rodents and shrews and further proved the role of these animals as the natural reservoirs of *Y. pestis* once survived plague infection in the study area. Detection of such antibodies in 35/199 (17.6%) (95% CI 12.57–23.6%) animals and 25/199 (12.6%) (95% CI 8.30–17.98%) when anti-mouse antibody and anti-rat antibody respectively were used as secondary antibodies, suggests that anti-mouse immunoglobulin is the most cross-reactive to the heterologous antibodies in the rodent population. These results are consistence with those obtained by Nakamura et al. [15]. In a similar study conducted in California, Smith et al. [25] detected *Y. pestis* antibodies in 18% of rodents by ELISA tests. By using similar tests (ELISA), Kilonzo et al. revealed that 11% rodents and 2% domestic dogs in Northern Tanzania had anti-plague antibodies [26].

The current studies further suggest that *M. natalensis* and *Crocidura* spp. were the highest anti-plague positive animals when anti-mouse antibody was used as secondary antibodies, suggesting their strong involvement as plague reservoirs in the area. These observations are consistence with those reported by [22, 27] that *M. natalesis* in Tanzania were the most plague-positive rodents for both IgG and IgM (10.6 and 9.2% respectively). In Mbulu district, Tanzania, it was observed that 60% of *Crocidura* spp. were positive for *Y. pestis* antibodies when tested using ELISA [27].

The detection of specific antibodies against Fra1 antigen of *Y. pestis* in domestic dogs indicates that the animals had been exposed to the bacteria within their movement range. According to literature, however, anti-plague antibodies in small carnivores remain detectable up to 2 years [28]. It is, therefore, likely that the animals came into contact with the infected materials (e.g. tissues of rodents, shrews and hares) which domestic dogs can easily kill and or feed on. The current results are consistent with those reported by Kilonzo et al. [26]. Similarly, in other studies, dogs were reportedly sero-positive for the anti-plague antibodies in Zimbabwe [29] and Democratic Republic of Congo [30]. Since dogs are very mobile it is most likely that they may have been infected either at the villages of their residence or in neighbouring villages. Dogs are generally considered to be suitable sentinel animals and hence, the presence of plague IgG antibodies in such animals is an indication that the latter had been in contact with *Y. pestis* either recently or recent past. The relationship of the dog to man and the dog’s rapid serologic response to the plague bacillus suggest that these animals could serve as useful amplifiers and/or sentinel for the detection of plague in areas frequented by man [30, 31].

The observation that 29/37 (78.3%) of rodent fleas collected were hosted by *M. natalensis* and that all the fleas collected from rodents were *Xenopsylla* spp. suggests that *M. natalensis* are the most favourite hosts of efficient plague vectors in the area. Such results are similar to those reported by Kilonzo et al. [26], that *M. natalensis* had the highest flea infestation rate (18%) followed by *Rattus rattus* (12%) in a plague endemic area in Northern Tanzania. The SFI in the area was very low (0.19), a fact which probably explains why there have not been any reported human plague cases in the area since the last plague outbreak. Indeed, it has been well documented that a rat-flea index of greater than one increases potential risk for plague infection of humans and an increase in the flea population in a plague endemic area may result in outbreaks of the disease [26, 32]. The rise in the flea population may be caused by climatic conditions such as temperature, rainfall and relative humidity. These have direct effect on development, survival, behaviour and reproduction of fleas and their populations [24]. An SFI of at least 0.5–1 is considered sufficient to maintain plague in a locality [33] and an index greater than one is reported to represent a potentially dangerous situation with respect to plague risk [34]. In the current study, however, the SFI was too low to results in the disease outbreak but gives useful information for the surveillance of plague and serves as an indicator of potential plague transmission [17, 32]. Elsewhere, a rat-flea index ranging from 1.3 to 3.7 was reported in a plague endemic focus when the disease was prevalent [35].

## Conclusions

The detection of plasminogen activator gene (Pla) of *Y. pestis* in rodents and shrews, and specific antibodies against *Y. pestis* F1 antigen in domestic dogs, rodents and shrews indicates that *Y. pestis* is still present in the study area and was similar to other *Y. pestis* bacterium elsewhere. These animals could also act as the sentinel to alert the community to the condition of *Y. pestis* in the area.

## Abbreviations

PCR: Polymerase chain reaction
Yp pla: *Yersinia pestis* plasminogen activator
Fra: Fraction gene
ELISA: Enzyme-linked immunosorbent assay
